# ChatGPT-4’s Level of Dermatological Knowledge Based on Board Examination Review Questions and Bloom’s Taxonomy

**DOI:** 10.2196/74085

**Published:** 2025-08-07

**Authors:** Hansen Tai, Carrie Kovarik

**Affiliations:** 1 SUNY Upstate Medical University, Syracuse, United States; 2 Department of Dermatology, Perelman School of Medicine, University of Pennsylvania, 3600 Spruce Street, 2 Maloney Building, Philadelphia, 19146, United States, 1 2156626597, 1 2153495615

**Keywords:** ChatGPT, dermatology, education, board exam, residency

## Abstract

Our study demonstrated the ability of ChatGPT-4 to answer 77.5% of all sampled text-based board review type questions correctly. Questions requiring the recall of factual information were answered correctly most often, with slight decreases in correctness as higher-order thinking requirements increased. Improvements to ChatGPT’s visual diagnostics capabilities will be required before it can be used reliably for clinical decision-making and visual diagnostics.

## Introduction

ChatGPT, a multimodal language model capable of answering multiple choice questions, incorporates visual inputs in its latest version, GPT-4. Lewandowski et al [1] recently assessed ChatGPT-3.5 and ChatGPT-4’s performance in dermatology examinations, finding that ChatGPT-4 significantly outperformed its predecessor, achieving over a 60% pass rate overall and >84% accuracy on photo-based questions. Building on this, our study classified ChatGPT-4’s correctly answered question types using Bloom’s taxonomy for cognitive complexity [2].

## Methods

We evaluated ChatGPT-4’s capabilities on the Basic, Core, and Applied examination questions from Dermatology-In-Review, an online dermatology board review preparation course. The Basic examination is a required examination for first-year US dermatology residents and tests dermatology fundamental knowledge. The Core and Applied examinations are taken late in residency and after residency, respectively. These tests examine more advanced clinical knowledge and focus on higher-order thinking. In total, 167 Basic, 210 Core, and 166 Applied multiple-choice questions without photos were formatted and fed into ChatGPT-4 using an algorithm in Python’s Pandas. ChatGPT-4’s in-depth responses to each query were captured, reviewed, and independently confirmed and coded as correct or incorrect (Table 1).

**Table 1.** ChatGPT-4 cases correct by testing category.^a^

	Correct	Incorrect	% Correct	Remember type questions: Correct %, Total %
Basic	139	28	83.20%	71/82 (86.6%), 82/167 (49.1%)
Core	158	52	75.20%	52/66 (78.8%), 66/210 (31.4%)
Applied	123	43	74.10%	35/46 (76.1%), 46/166 (27.7%)

a *P* value=.0382, Pearson’s Chi-squared test for the Basic versus Core+Applied Examinations.

We categorized text-based questions according to Bloom’s taxonomy using a Python function. One author (CK) and ChatGPT-4 categorized each question into a specific category of Bloom’s Taxonomy using guidelines [2]. In the case of a discrepancy, ChatGPT-4’s reasoning for the decision was considered, which assisted in the reconciliation of categorization. Bloom’s categories included Remember (includes lower-level thinking, such as knowledge and comprehension), Apply, Analyze, Evaluate, and Synthesize. All statistics were performed using R statistical software, including the Pearson chi-squared test (Table 1) and Fisher exact test (Table 2).

Photo-based questions were entered directly into ChatGPT-4, along with structured messages and answer choices, and responses were recorded. Fifty-three photo cases from all board categories were used.

**Table 2.** ChatGPT-4 cases correct by Bloom category (all cases).^a^

	Correct	Incorrect	Total	% Correct
Remember	158	35	193	81.9%
Apply	168	51	219	76.7%
Analyze	56	19	75	74.7%
Evaluate	37	14	52	72.5%
Synthesize	1	3	4	25.0%
Total	420	122	542	77.5%

a *P* =.059, Fisher exact test.

## Results

Overall, ChatGPT-4 answered 77.5% of all sampled text-based questions correctly. Varying levels of accuracy were demonstrated in answering board questions within different Bloom categories. In the “Remember” category, the model correctly answered 158/193 (81.9%). “Remember” is considered the most basic level of educational understanding, with the ability to recall or comprehend information without applying the concept [3]. ChatGPT-4 performed the best in this category; however, it did significantly (*P*=.0382) better on the “Remember” questions from the Basic examination compared to those on the Core and Applied sections combined (Table 1). As the Bloom categories progress from Apply to Analyze, Evaluate, and Synthesize, a solid foundation of knowledge and higher-order thinking is necessary. Table 2 demonstrates a decreasing trend (*P*=.059) in the percent correctness for the ChatGPT-4 answers moving from “Remember” to the classes of higher-order thinking.

Of the 53 questions, 18 (34%) with photos were answered correctly, with none of the “What is the histologic diagnosis?” question stems answered correctly. Excluding these, 18/38 (47.3%) had the correct answer. Notably, photo questions with leading information were more likely to be given the correct response.

## Discussion

ChatGPT-4 correctly answered 77.5% of all text questions correctly, similar to the results of Lewandowski et al [1], in which ChatGPT-4 answered 80.7%‐84% of the questions correctly on English-based Dermatology assessments. Our outcomes differed in that they were able to obtain a much higher number of correct responses on photo-based questions compared to our study, where ChatGPT-4 was only able to answer approximately one-third of the photo cases correctly. Hirosawa et al [4]assessed the impact of adding image data to clinical textual data on ChatGPT-4’s diagnostic accuracy. They found that integrating image data into ChatGPT-4 did not significantly enhance diagnostic accuracy, and it predominantly relies on textual data, limiting its ability to use the diagnostic potential of visual information fully [[4]]. This corroborates our findings of poor analysis of photo cases and improved correctness when leading question stems were given.

Overall, our study demonstrates the ability of ChatGPT-4 to answer text-based questions from Dermatology-In-Review at a high level. Questions requiring the recall of factual information were answered correctly most often, with slight decreases in correctness as higher-order thinking requirements increased. Improvements to ChatGPT-4’s visual diagnostics capabilities will be required before it can be used reliably for visual interpretation and clinical decision-making. In its current state, ChatGPT-4 may be used as an educational tool for students and trainees when exploring core factual knowledge; however, trainees and practitioners should not rely on ChatGPT for higher level inquiries, such as analyzing clinical scenarios or image interpretation.

Our study has several limitations. Bloom’s taxonomy is a continuum, and question classification can be complex. We used board review questions, and this may not be generalizable to true board questions. The edition of ChatGPT-4 used in this study had been trained with data only up to December 2023 [5].
